# Archaeome structure and function of the intestinal tract in healthy and H1N1 infected swine

**DOI:** 10.3389/fmicb.2023.1250140

**Published:** 2023-09-13

**Authors:** Alexander Meene, Laurin Gierse, Theresa Schwaiger, Claudia Karte, Charlotte Schröder, Dirk Höper, Haitao Wang, Verena Groß, Christine Wünsche, Pierre Mücke, Bernd Kreikemeyer, Martin Beer, Dörte Becher, Thomas C. Mettenleiter, Katharina Riedel, Tim Urich

**Affiliations:** ^1^Institute of Microbiology, University of Greifswald, Greifswald, Germany; ^2^Friedrich-Loeffler-Institut, Greifswald, Germany; ^3^Institute for Medical Microbiology, Virology and Hygiene, Rostock University Medical Centre, Rostock, Germany

**Keywords:** methanogenesis, intestinal tract microbiome, methanogenic archaea, 16S rRNA gene sequencing, metaproteomics, metatranscriptomics

## Abstract

**Background:**

Methanogenic archaea represent a less investigated and likely underestimated part of the intestinal tract microbiome in swine.

**Aims/Methods:**

This study aims to elucidate the archaeome structure and function in the porcine intestinal tract of healthy and H1N1 infected swine. We performed multi-omics analysis consisting of 16S rRNA gene profiling, metatranscriptomics and metaproteomics.

**Results and discussion:**

We observed a significant increase from 0.48 to 4.50% of archaea in the intestinal tract microbiome along the ileum and colon, dominated by genera *Methanobrevibacter* and *Methanosphaera*. Furthermore, in feces of naïve and H1N1 infected swine, we observed significant but minor differences in the occurrence of archaeal phylotypes over the course of an infection experiment. Metatranscriptomic analysis of archaeal mRNAs revealed the major methanogenesis pathways of *Methanobrevibacter* and *Methanosphaera* to be hydrogenotrophic and methyl-reducing, respectively. Metaproteomics of archaeal peptides indicated some effects of the H1N1 infection on central metabolism of the gut archaea.

**Conclusions/Take home message:**

Finally, this study provides the first multi-omics analysis and high-resolution insights into the structure and function of the porcine intestinal tract archaeome during a non-lethal Influenza A virus infection of the respiratory tract, demonstrating significant alterations in archaeal community composition and central metabolic functions.

## 1. Introduction

The intestinal tract, known as one of the environments with a very high microbial diversity, provides a habitat for a broad variety of different prokaryotic microorganisms. Bacterial genera consuming monomeric and polymeric carbohydrates, e.g., *Lactobacillus* and *Prevotella* as well as short chain fatty acid (SCFA) producers like *Ruminococcus, Lachnospira*, and *Bacteroides* are the predominant commensals of the porcine intestinal tract (Holman et al., 2017; Gierse et al., 2020). The less abundant methane producing prokaryotes, exclusively from the archaeal domain, play an important role in the digestive tract of pigs and many vertebrates (Youngblut et al., 2021). The removal of H_2_ and CO_2_ during polysaccharide decomposition is related to methanogenic archaea and positively affects the fermentation rate (Stams, 1994) and energy acquisition for the host (Samuel and Gordon, 2006; Armougom et al., 2009; Patil et al., 2020; Deng et al., 2021). Predominant methanogenic species in this highly complex system were identified with archaeal-specific 16S rRNA gene primers as *Methanobrevibacter smithii*, *Methanobrevibacter millerae* and *Methanosphaera cuniculi* (Luo et al., 2012). In addition to these previously-described species, Mi et al. (2019) identified members of the order *Methanomassiliicoccales* (with appr. 15% relative abundance) and *Methanomicrobiales* (with less than 5% relative abundance) in the colonic digesta of finishing pigs through methyl coenzyme-M reductase (*mcrA*) gene analysis.

In addition to natural sources of methane emission including wetlands, biomass burning and termites, human activity such as livestock production and rice cultivation constitute a major source of global methane (Saunois et al., 2020). Due to the increase in global meat and dairy production (Ritchie and Roser, 2017), enteric methane emissions (EME) should be considered a major contributor of greenhouse gas emissions from livestock (Thorpe, 2009; Patra, 2014) with a high potential for mitigation (Herrero et al., 2016).

Methane from livestock is mainly a by-product in ruminants and mono-gastric animals from plant carbohydrate decomposition (Monteny et al., 2001). Methanogenesis can be divided into three substrate-differentiated pathways: 1. Hydrogenotrophic methanogenesis [CO_2_-reduction to methane with H_2_ as electron donor (Liu and Whitman, 2008)] represented by archaea belonging to the order *Methanobacteriales* [e.g., *Methanobrevibacter* (Miller et al., 1982; Rea et al., 2007)] and *Methanococcales* (Goyal et al., *2016*). 2 Methylotrophic methanogenesis (reduction of methylated compounds such as methanol and methylamines to methane) by members of the *Methanomassiliicoccales* (including the *Methanomethylophilaceae* family), as well as *Methanosphaera* species (Miller et al., 1982; Reeve et al., 1997; Dridi et al., 2012). 3. Acetoclastic methane formation (in which methane is produced by acetate with carbon dioxide as a byproduct). In particular, archaea from the order *Methanosarcinales* use the acetoclastic pathway, which is the source of two-thirds of biogenic methane emissions (Lyu et al., 2018).

The initial step all methanogens have in common is the reduction of methyl-coenzyme M to CH_4_ through methyl-coenzyme M reductase (*mcr*). In addition to *mcr*, the reduction of the methyl-CoM complex to CH_4_ requires a second co-enzyme, named CoB (Reeve et al., 1997). These two limiting steps in methanogenesis are both regulated by the *mcrA* gene, which can therefore be targeted as a marker gene for the quantification of all methanogens present in human, animal and environmental samples (Ufnar et al., 2007; Scanlan et al., 2008; Mi et al., 2019; Weil et al., 2020).

In swine, methanogenesis primarily occurs in the lower (colonic) gut sections (Robinson et al., 1989; Jensen, 1996). No methane is produced in the stomach or small intestine, and only low concentrations were found in the cecum and proximal colon by Jensen and Jørgensen (1994). Furthermore, this study described an approximate nine-fold increase of *in vivo* methane production for high fiber diet (12.5 liters/day) compared to a low fiber diet (1.4 liters/day) in 7-month-old pigs, as well as a positive correlation for quantitative *in vivo* and *in vitro* gas measurements. Changes in the taxonomic composition of methanogenic archaea were observed during fibrous supplementation of pea fiber (Luo et al., 2017). In addition to diet, methanogen activity is also chemically limited by the redox potential (Eh). Mi and colleagues described a positive correlation between the *Methanobacteriales* population and the Eh (Mi et al., 2019). While dietary and chemical implications on the community of methanogenic archaea in the porcine intestinal tract are investigated to some extent, the impact of pathogen infection onto the swine archaeome is to our knowledge unknown and should be addressed to fulfill the knowledge on this metabolic important group.

For instance, swine are natural hosts for influenza A virus (IAV) and are susceptible to infection, with the human and avian IAV strains serving as ´mixing vessel´ (Kida et al., 1994). Because coinfection of the strains can lead to genetic reassortment (Scholtissek, 1990), new influenza strains often originate in swine (Ma et al., 2008, 2009). Therefore, a porcine model is crucial to evaluate as a new biomedical animal model for human influenza research (Rajao and Vincent, 2015; Schwaiger et al., 2019). Porcine models are particularly useful due to their anatomical and physiological similarity to humans (Pang et al., 2007; Spurlock and Gabler, 2008). Crucially for pathogenesis, the distribution of IAV receptors between the porcine and human respiratory tract are highly similar (Shinya et al., 2006).

Influenza virus infections represent a large burden for both animal and human holobionts that potentially affect the manifold interactions between the host, its microbiome and within the microbiome. Methanogenesis in the intestinal tract is a microbiome-host interaction with benefits for the host that could be influenced by a respiratory tract infection with the influenza A virus.

In this study, the intestinal archaeome of pigs was investigated with a multi-omics approach to (1) provide high-resolution insights into the structural and temporal dynamics of methanogens along the pig intestinal tract (ileum-colon-feces), (2) reveal the active methanogenesis pathways of core methanogens and (3) analyze the impact of a non-pathogenic IAV infection onto the archaeome. For this purpose, we investigated recently published multi-Omics data of an animal trial (Gierse et al., 2021) and complemented them with a metatranscriptomics approach.

## 2. Materials and methods

### 2.1. Animal study design

All samples for this study were provided by the Department of Experimental Animal Facilities and Biorisk Management of the Friedrich-Loeffler-Institut on the Isle of Riems within the H1N1pdm09 animal experiment under reference number 7221.3–1-035/17 (Schwaiger et al., 2019). For this study, 16 influenza virus A/Bayern/74/2009-infected and three mock-infected (serving as experimental control) German landrace pigs, which were 7 weeks of age at the beginning of the study, were analyzed. The sampling scheme is described in Table 1. Swine fecal samples were collected within 30 s of defecation for a period of 31 days to account for longitudinal shifts. Digesta from intestinal sections (IS) (ileum, proximal and distal colon from three healthy and three infected individuals) were sampled during necropsy on days 7 (*n* = 1), 25 (*n* = 2) and 30 (*n* = 3). All samples were immediately frozen on dry ice and subsequently stored at – 80°C.

**Table 1:** Longitudinal sampling scheme and number of samples per sampling day for 16S rRNA, metaproteomic and metatranscriptomics analysis.

Individual number of analyzed samples after homogenization		Days after starting point										
		0	2	4	7	14	21	22	23	25	31	IS
16S rRNA gene sequencing	Healthy pigs (*n* = 3)	3	2	3	3	2	3	3	3	3	3*	9
	Infected pigs (*n* = 16)	14	16	16	16	11	12	8	8	8	4	8
Metaproteomics	Healthy pigs (*n* = 3)	3	–	2	3	–	3	–	–	3	3*	–
	Infected pigs (*n* = 16)	3	–	3	3	–	3	–	–	3	–	–
Metatranscriptomics	Healthy pigs (*n* = 3)	3	–	3	–	–	3	–	–	–	3*	–
	Infected pigs (*n* = 16)	7	–	4	–	–	–	–	–	–	2	–

^*^Samples from healthy animals were collected on day 30.

### 2.2. Sample processing

For 16S rRNA gene sequencing and metaproteomics approach, fecal samples and intestinal sections (IS) were homogenized by the Covaris® CP02 CryoPrep™ instrument (Covaris Ltd., Brighton, United Kingdom) and processed as previously described in Gierse et al. (2020).

### 2.3. DNA extraction, 16S rRNA gene amplicon library preparation, sequencing, and bioinformatic processing

Nucleic acids were extracted from appr. 100 mg fecal powder using a bead-beating phenol chloroform extraction protocol (Berry et al., 2012) with subsequent precipitation of nucleic acids with 3 M Na-acetate and isopropanol as described in Gierse et al. (2020). Afterwards, the DNA pellets were washed with 70% v/v ethanol before being dried and re-suspended in DEPC-treated MilliQ water for downstream applications. The DNA content was quantified with Qubit™ dsDNA Broad Range Assay Kit (Invitrogen™). Next, the 16S rRNA amplification and Illumina MiSeq sequencing were performed using the V4 primer pair 515F (5`-GTG-YCA-GCM-GCC-GCG-GTA-A-3`) /806R (5`-GGA-CTA-CNV-GGG-TWT-CTA-AT-3`), as described in Gierse et al. (2020). The Illumina MiSeq resulted in an average output per sample from around 100,000 reads per sample and the sequences were submitted to European Nucleotide Archive (ENA) under the project name “Influence of an Influenza-A-Virus infection on the respiratory and gastrointestinal tract microbiome and archaeal community composition’ (project number PRJEB42450, accession number ERP126308).

Analysis was performed in R (R Core Team, 2020) using the dada2 pipeline package version 1.11.1 (R-version 3.6.1) for sequence annotation. Advanced bioinformatics processing (e.g., alpha-and beta diversity, Bray–Curtis dissimilarity and nonmetric multidimensional scaling) was done in R with packages “vegan,” “ggplot,” “phyloseq,”” plyr” and“reshape2” as described in Gierse et al. (2020). For statistical analysis, Kruskal-Wallis rank sum tests were performed (value of *p* ≤0.05) and the *p* values were corrected using the Benjamini and Hochberg false discovery rate (fdr) approach in R.

### 2.4. Protein extraction, mass spectrometry, database assembly and data analysis

Metaproteomic analysis was performed as previously described in Gierse et al. (2020). Proteins were extracted from appr. 100 mg fecal powder using the TRIzol-based extraction protocol, and protein concentrations were measured via Pierce BCA Protein Assay (Smith et al., 1985; Kessler and Fanestil, 1986; Wiechelman et al., 1988; Brown et al., 1989). Subsequently, 30 μg protein were loaded on a 4–22% Criterion TGX precast gel (BioRAD, Hercules, CA, United States) and stained with Colloidal Coomassie Brilliant Blue G-250 (Neuhoff et al., 1988). Each lane was cut in 10 fractions, and each fraction was processed into smaller pieces, de-stained and purified according to the ZipTip manufacturer’s protocol (C18, Merck Millipore, Billerica, MA, United States). Peptide-containing solution was eluted in glass vials and vacuum centrifugation was performed until dry. Finally, the peptides were re-suspended in 10 μL of 0.1% (v/v) formic acid for mass spectrometric analysis as previously described (Gierse et al., 2020). Protein identification was performed using Mascot Daemon version 2.6.2 (Matrix Science Ltd., London, United Kingdom) against a custom archaea-specific database, which was built using the results of the 16S rRNA gene sequencing data from the corresponding fecal samples. The database contained all UniprotKB entries (access date: 09.06.2020) for every identified archaeal member of the gastrointestinal microbiome. Because *Clostridium* sp. CAG_221, *Lactobacillus reuteri* and *Prevotella copri* were identified as the most abundant species in the porcine GIT microbiome, these species were included and used as a reference for bacterial-originated proteins. Furthermore, all entries for *Sus scrofa* and influenza A virus were included in the database. Compared to a database including all members of the porcine gastrointestinal microbiome, the amount of archaeal originated protein groups was increased from ~0.4% to ~4% of all identified protein groups through use of the reduced database.

For validation of Mascot data, Scaffold 4.8.7 and X!Tandem (version X! Tandem Alanine, 2017.2.1.4) were used. Metaproteome annotation pipeline Prophane^1^ was employed for taxonomic and functional protein analysis (Schiebenhoefer et al., 2020) with the same parameters as previously described (Gierse et al., 2020). The mass spectrometry proteomics data were deposited to the ProteomeXchange Consortium via the PRIDE (Perez-Riverol et al., 2019) partner repository with the data set identifiers PXD044365.

### 2.5. RNA extraction, cDNA synthesis, high-throughput sequencing, and metatranscriptomic analysis

The analyzed samples were provided from the same infection trial (reference number 7221.3–1-035/17; Schwaiger et al., 2019) in which swine were infected with influenza virus A H1N1pdm09. Fecal and rectal swab samples from the swine were collected on days 0 (day of first infection), 4, 21 (day of second infection) and finally day 30 (control group) or 31 (infection group).

The preparation of the samples for sequencing was performed as published (Wylezich et al., 2018) with a few modifications. Prior to RNA extraction, the fecal samples were diluted threefold (2 g of feces and 4 mL water) and homogenized using the IKA Ultra Turrax Tube Drive System (IKA, Staufen, Germany) in an ST-20 Tube with a stirring device of 5 steel balls (5 mm) to ensure representative subsamples. Subsequently, the coarse fiber components were separated from the homogenates with single-use nylon sieves (mesh size 1 mm; Carl Roth, Karlsruhe, Germany). The rectal swab samples (Rayon and Polyester Dryswabs™, Medical Wire and Equipment, England) were incubated *via* shaking in 1 mL distilled water at 4°C and 1,400 rpm for 4 min (ThermoMixer®, Eppendorf, Wesseling-Berzdorf, Germany). The supernatant was then used for RNA extraction (see below).

To extract the nucleic acids, 200 μL of the feces homogenates or 250 μL of the swab supernatants were disintegrated using cryoPREP (Covaris Inc., Woburn, United States). The pulverized material from feces homogenates or swab supernatants was suspended in 800 μL or 1 mL AL lysis buffer (Qiagen, Hilden, Germany), respectively, and preheated to 56°C. Subsequently, RNA was extracted from the lysate using TRIzol Reagent (Thermo Fisher Scientific Inc., Waltham, United States) in combination with RNeasy Mini Kit (Qiagen) including on-column DNase digestion (Qiagen) according to the manufacturer’s instructions. The RNA was reverse-transcribed into cDNA with the cDNA-Synthesis System (Roche, Mannheim, Germany) and hexanucleotide primers (Roche). After fragmentation with a M220 Focused-ultrasonicator (Covaris), Ion-Torrent compatible DNA libraries were prepared using IonXpress barcode adapters (Life Technologies, Darmstadt, Germany) and a GeneRead DNA Library L Core Kit (Qiagen) according to the manufacturer’s instructions. Thereafter, size exclusion, quality control with a Bioanalyzer 2,100 and a High Sensitivity-Chip (Agilent Technologies, Santa Clara, CA, United States), and library quantification using the KAPA Library Quantification Kit (Roche) were performed. Deep sequencing of pooled sequence libraries was completed with Ion 530 Chips in 400 Bp mode on an Ion Torrent S5 XL instrument (Thermo Fisher Scientific Inc.) according to the manufacturer’s instructions.

Resulting RNA sequences were submitted to European Nucleotide Archive (ENA) under the project name “Influence of an Influenza-A-Virus infection on the respiratory and gastrointestinal tract microbiome and archaeal community composition’ (project number PRJEB42450, accession number ERP126308).

RNA sequences were quality-checked with FastQC and subsampled into rRNA and non-rRNA fractions using SortMeRNA (Kopylova et al., 2012). The resulting non-rRNA from all samples were pooled and aligned against the NC_nr database (accessed 12/03/2020) using DIAMOND (Buchfink et al., 2015). Taxonomic and functional analysis was performed in MEGAN6 Ultimate using the lowest common ancestor (LCA) algorithm (min score 50; top percent 4; min support 1; Huson et al., 2016). Taxonomic binning of the mRNA reads was done to the phylum Archaea, family *Methanobacteriaceae* and the genera *Methanobrevibacter* and *Methanosphaera*, respectively. Within these taxonomic bins, mRNA sequences assigned to the KEGG category “methane metabolism” were considered as methanogenesis transcripts of the respective taxa (Kanehisa and Goto, 2000).

## 3. Results and discussion

### 3.1. Alpha diversity and archaeal community composition in gut sections and feces

**Figure 1:**
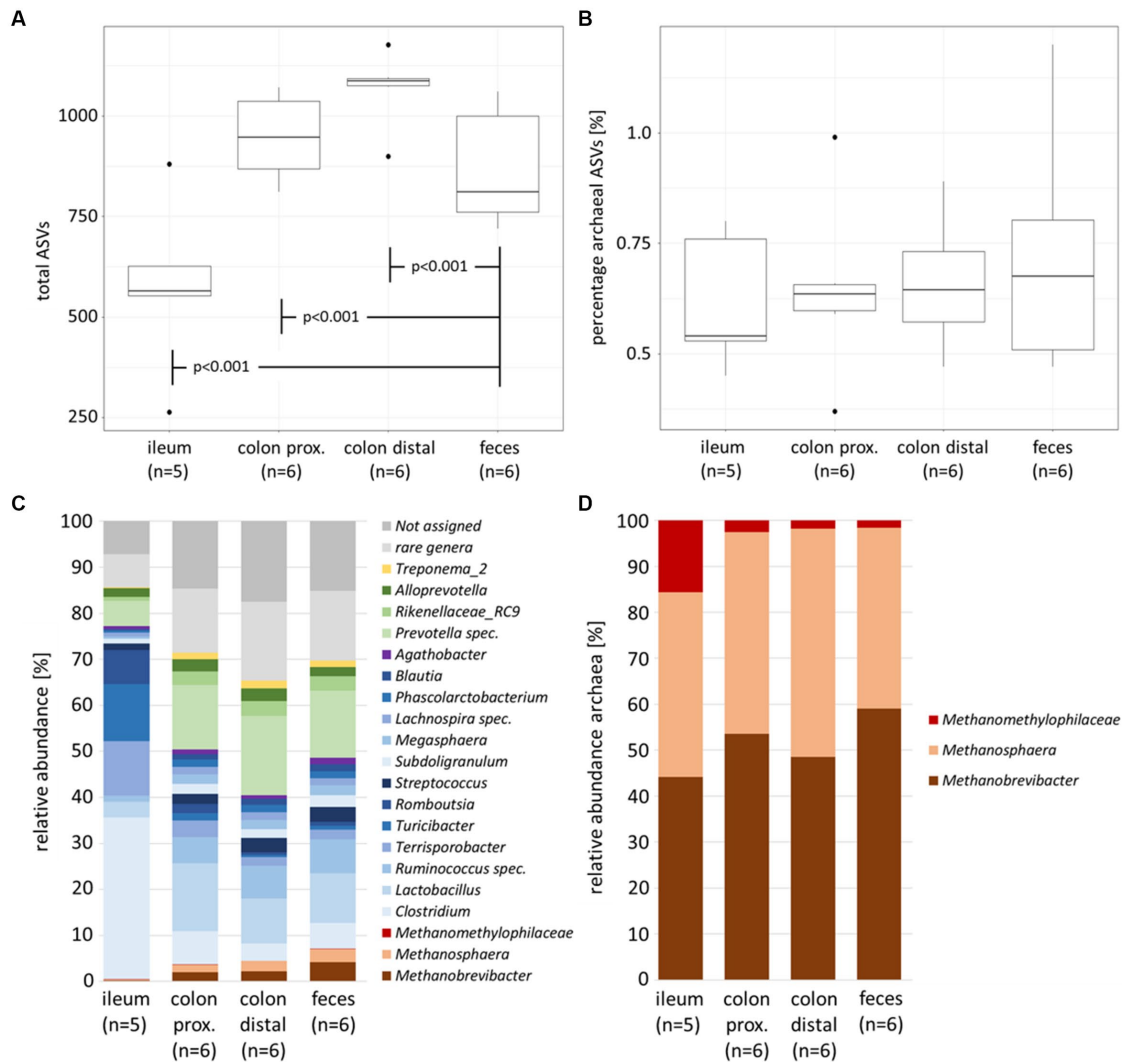
Alpha diversity parameters and community composition of the porcine intestinal tract (naïve and infected individuals summarized). Boxplots with total number of ASVs **(A)** and percentage of archaeal ASVs **(B)** between sample types. Barplots with total prokaryotic (bacterial and archaeal) community composition **(C)** and archaeal community composition **(D)**. Color-coding represents the phylum affiliation, with Firmicutes in blue color grades, Bacteroidetes in green, Euryarchaeota in brown and Spirochaetes in yellow. Bacterial genera below 1% relative abundance were excluded and all present archaeal genera remained in the dataset.

The microbial richness of the total prokaryotic community between the feces of naïve and infected animals demonstrates no significant difference, while the microbial diversity within the same experiment showed significant alterations in the high abundant families *Prevotellaceae*, *Clostridiaceae* and *Lachnospiraceae* (Gierse et al., 2021). In contrast, the comparison between feces and intestinal sections (summarized within naïve and infected individuals) indicated significant differences (*p* < 0.001). The number of identified taxa (represented by the amplicon sequence variant ASV) was highest in the colonal sections (avg. colon proximal 947 ASVs and colon distal 1,069 ASVs) and lowest in ileum (avg. 577 ASVs) with significant differences to intermediate fecal data (avg. 868 ASVs) (Figure 1A).

The percentage of archaeal ASVs was highest in fecal samples (avg. 0.72%), with negligible lower amounts in the three different intestinal samples (colon distal avg. 0.66%, colon proximal avg. 0.65% and ileum avg. 0.62%; Figure 1B). Although the colonel sections had higher rates of prokaryote colonization, the relative number of archaea was rather similar between the intestinal samples.

A deeper look into the gastrointestinal compartments in Figure 1C revealed Firmicutes and Bacteroidetes as predominant phyla. This is consistent with the previously published study on the healthy cohort of an animal trial (Gierse et al., 2020) as well as other studies (Holman et al., 2017; Le Sciellour et al., 2019). Additionally, Spirochaetes and Euryarchaeota were found in the intestinal tract. The small intestine, represented by the ileum, was colonized by members of the genera *Clostridium*, *Terrisporobacter* and *Romboutsia*, whereas the compartments of the large intestine and feces are primarily dominated by *Lactobacillus*, *Ruminococcus* and *Prevotella spec.* These intestinal section-dependent shifts were also observed within the archaeal community composition. While the relative abundances of the three dominant archaeal genera in the small intestine (*Methanobrevibacter*, *Methanosphaera* and *Methanomethylophilaceae*) were below 0.5%, between 4 and 7% of these members were found in the colonal sections and feces. The overall increasing relative abundance in methanogenic archaea in the biological course of the intestinal tract was characterized by a significant increase of *Methanobrevibacter* (*p* < 0.05) and *Methanosphaera* (*p* < 0.05), especially between the ileum and feces. In contrast, *Methanomethylophilaceae* showed stable but very low relative abundances ranging from 0.075% up to 0.1% along the natural course of the intestinal tract.

Within the archaeal community, the relative abundance of *Methanobrevibacter* increases significantly (*p* = 0.028) from 44.2% in the ileum to 59% in feces (Figure 1D). A possible consequence of this shift is that methane formation via hydrogenotrophic CO_2_ reduction increases relatively along the natural course of the intestines. In a recent study, Mi et al. (2019) sequenced *mcrA* gene amplicons and identified similar abundances of colonic digesta of Chinese pigs, with a *Methanobrevibacter* abundance 57%. In our study, methylotrophic *Methanosphaera* were present in 40.2% of the ileum samples, 46.8% of colonic sections (44% proximal and 49.7% distal) and 39.3% of fecal samples. Although the differences between the intestinal sections were insignificant at the 0.05 threshold, one could assume higher rates for methylotrophic methanogenesis in the large intestines compared to fecal samples. Mi et al. (2019) described a lower relative abundance for *Methanosphaera* of 14% and a higher relative abundance of *Methanomassiliicoccales* of 15%.

We further investigated this longitudinal dynamic in highly abundant genera in the dissimilarity analysis at the end of section 3.2.

### 3.2. Temporal dynamics of archaeal phylotypes in feces

**Figure 2:**
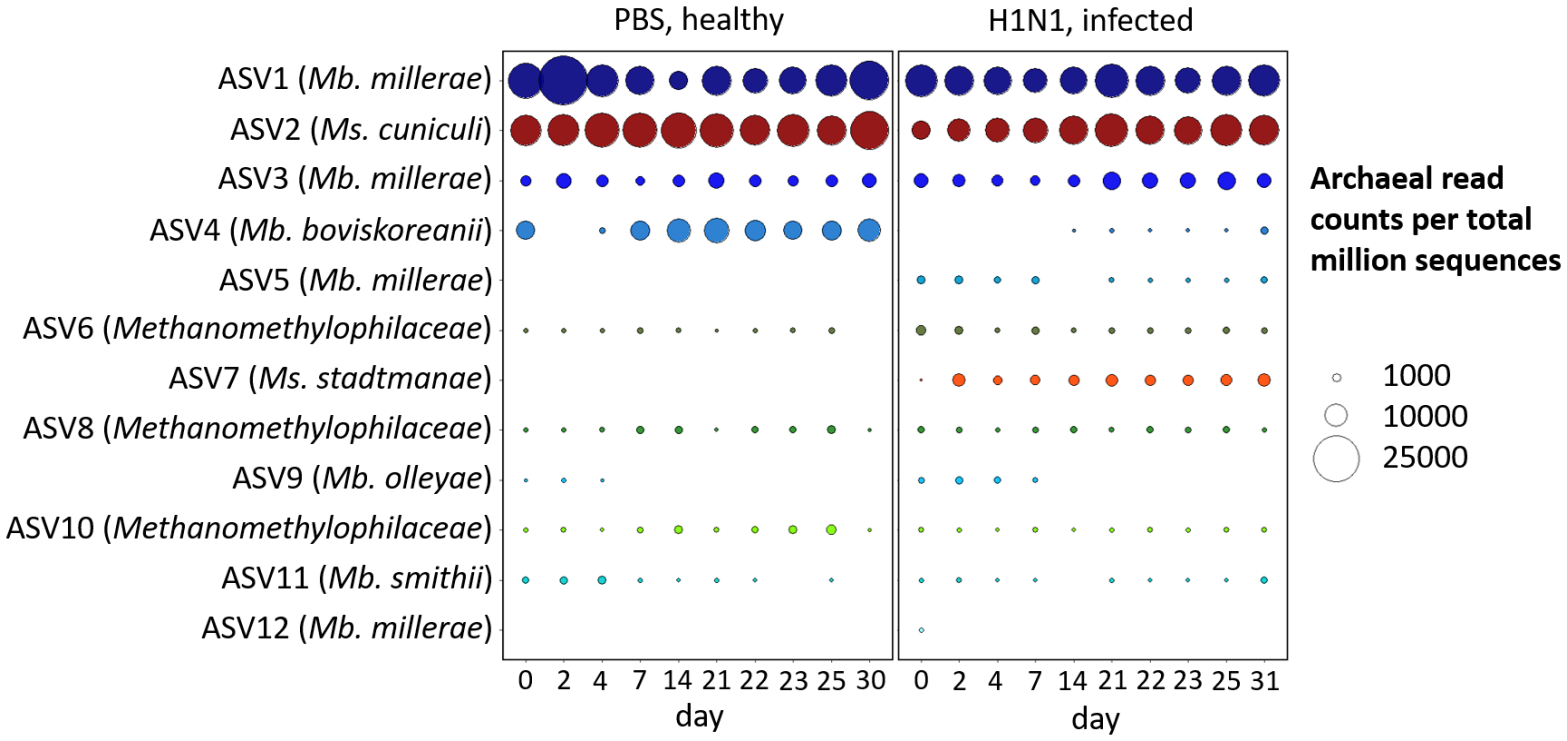
Temporal dynamics of archaeal phylotypes in feces of healthy and Influenza A virus-infected swine. Bubble plot of the 12 most abundant ASVs and corresponding Blast output to species level with highest sequence identity with *Mb.* = *Methanobrevibacter* and *Ms.* = *Methanosphaera*, and on family level with *Methanomethylophilaceae*. Bubble size indicates the absolute amount of archaeal read counts per million sequences (archaea and bacteria).

We have analyzed the temporal dynamics of the archaeal microbiome of pigs over the course of 1 month, which was a substantial part of the animal’s life span both in typical animal experiments (Kim et al., 2011; Le Sciellour et al., 2019; Mi et al., 2019; Wang et al., 2019), and livestock swine (Gutierrez et al., 2015). There were clear treatment-dependent differences between the archaeal community composition of healthy and influenza A virus-infected individuals (Figure 2).

The majority of ASVs in the porcine intestinal tract belong to the species *Methanobrevibacter millerae*, followed by *Methanosphaera cuniculi* and *Methanobrevibacter boviskoreanii*, together representing 80–90% of the archaeal community. In contrast to pigs, predominant phylotypes of the human intestines were *Methanobrevibacter smithii* and *Methanosphaera stadtmanae* (Miller et al., 1982; Dridi et al., 2009). As in our porcine model organism, *Methanosphaera cuniculi* was recently reported in human intestines (Chibani et al., 2022).

Initial structural and functional indications of a respiratory tract infection with influenza A virus on the gastrointestinal microbiome via metaproteome analysis were reported in a related study of Gierse et al. (2021). Microbial richness, bacterial diversity and central enzymes were significantly affected by the infection and indicate intestinal disturbances, most likely caused by the IAV infection. In our dataset from the same animal trial (Schwaiger et al., 2019; Gierse et al., 2021) the number of *Methanobrevibacter boviskoreanii* (ASV4) reads was significantly reduced by first Influenza A virus infection (d0, *p* < 0.01), whereas the naïve animals showed rather stable read counts. On the other hand, the unique ASV7, *Methanosphaera stadtmanae*, only occurred in the infected animals on day 2 post-infection and was absent in the naïve animals, which is possibly indicative of a treatment dependent effect (*p* < 0.001). Additional significant differences (*p* < 0.01) between the naïve and the H1N1 infected animals were observed for the predominant *Methanosphaera cuniculi* (ASV2), *Methanobrevibacter millerae* (ASV5) and within the family *Methanomethylophilaceae* (ASV6).

A phylogenetic analysis of methanogens in swine feces by Mao et al. (2011) indicated that the majority of present phylotypes belonged to *Methanobrevibacter* spp., followed by *Methanosphaera stadtmanae* and, with a lower percentage in sequence similarity, *Aciduliprofundum boonei*. The study of Guindo et al. (2021) identified *Methanobrevibacter smithii* as the most prevalent and *Methanobrevibacter millerae* as a minor dominant in the pig’s digestive tract *via* real-time PCR and PCR sequencing. Contrary to this study, we observed a minor prevalence for *Methanobrevibacter smithii* (ASV11) but identified *Methanobrevibacter millerae* (ASV1, ASV3, ASV5, and ASV12) as the most dominant phylotype in our dataset.

Similarly, Federici et al. (2015) described a strong significant shift from *Methanobrevibacter smithii* to *Methanobrevibacter boviskoreanii* due to weaning, which could be an explanation for the high abundance of *Methanobrevibacter boviskoreanii* and slightly decreasing occurrence of *Methanobrevibacter smithii*, particularly within the healthy pigs of our study.

The absence of *Methanosphaera stadtmanae* (ASV7), an H2-and methanol-dependent methanogen (Saengkerdsub and Ricke, 2014), in the healthy cohort (see Figure 2) could lead to the assumption of a decreased level of the methylotrophic methanogenesis pathway compared to the infected cohort. However, the predominant ASV2 *Methanosphaera cuniculi* was significantly higher in the healthy cohort, which could have a balancing effect on methane formation between these two groups.

#### 3.2.1. Dissimilarity analysis revealed treatment and sample type-specific clustering

**Figure 3:**
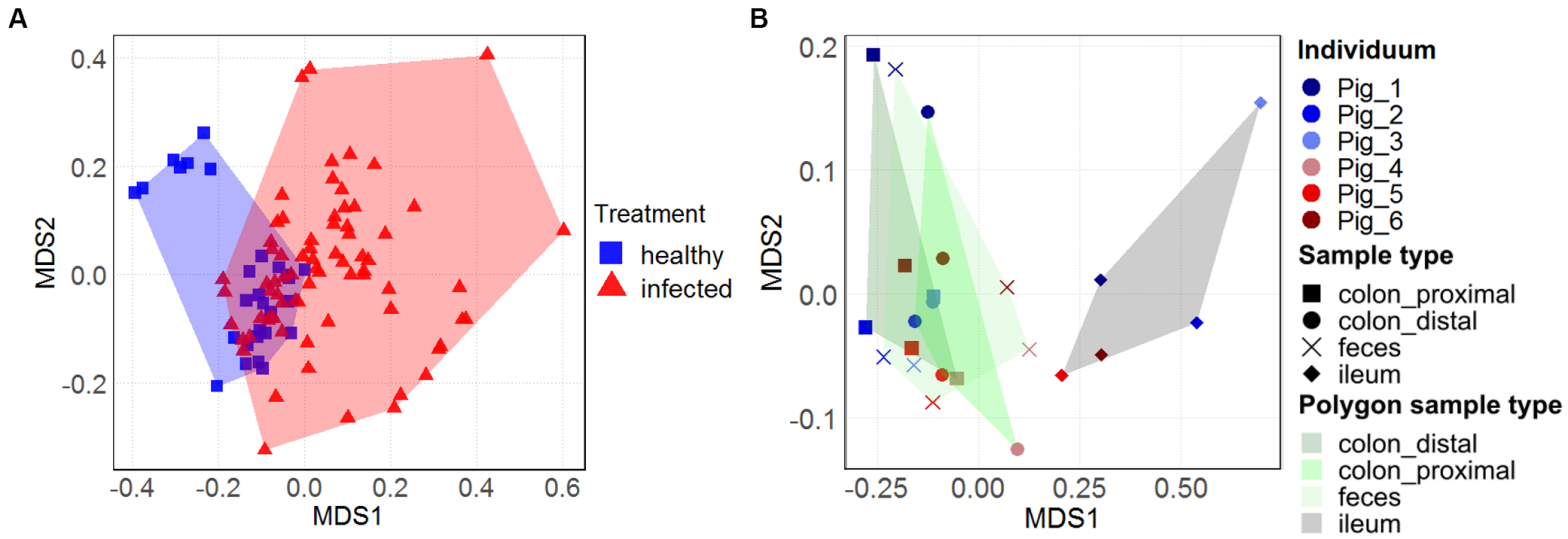
Temporal and longitudinal development of the archaeal community composition in swine feces and intestinal samples. Nonmetric multidimensional scaling (nMDS) plots, based on Bray-Curtis dissimilarities of the archaeal community composition over 31 days in feces on ASV-level [**(A)**, *n* = 19] and of different intestinal locations and feces from the individuals [**(B)**, *n* = 6]. Treatment in **(A)** and **(B)** is represented by the colors blue (healthy) and red (H1N1-infected). Sample type in **(B)** is represented by the shape and polygon areas highlighting the location-specific clustering. The ileal data points are located on the right, while the colonal and fecal data points are clustered on the left of the plot.

The overall view on the nonmetric multidimensional scaling (nMDS) plots (Figures 3A,B) reveals treatment-and sample type-specific clustering in the archaeal community composition. Only within the three healthy animals (represented by blue squares), two distinct sex-dependent clusters (female in the upper left area) could be seen (Figure 3A). However, within the larger infected cohort, there was no sex-dependent clustering. Although there are more infected animals in the dataset, the H1N1 infection seems to spread out the archaeal community composition of the intermediate (d7-d21) time points. There was a large spread between the community composition of the naïve animals with the H1N1 infected animals (*p* < 0.05). The majority of the healthy individuals cluster around the origin, except for the healthy female individual spreading to the upper left area.

Effects of sample location-specific clustering in the intestinal microbiome (Figure 3B), previously described in Gierse et al. (2020), was also apparent in the archaeal community composition. The datapoints of the fecal samples and the content of the colonal sections (distal and proximal) were in close proximity to one another (depending on sex, where the isolated upper left area represents the female animal). In contrast, the ileal data points cluster in the right area (*p* = 0.001). This clustering coincides with the observation of increased archaeal relative abundance in both groups (Figures 1C,D).

### 3.3. Functional analysis of the archaeal metatranscriptome and metaproteome

#### 3.3.1. Metatranscriptomics of fecal samples revealed dominant pathways in *Methanobrevibacter* and *Methanosphaera* spp.

Metatranscriptomic methods were used to identify the types of methanogenesis carried out by the gut archaea. Total RNA metatranscriptomes of healthy and infected individuals resulted in 746,485 mRNA transcripts, most of which originated from bacteria (97%). More than 12,500 mRNAs were classified as archaea (1.7%). Among those, the majority (12,067) were classified as belonging to *Methanobacteriaceae*. Functional annotation using the KEGG database revealed that approximately 50% of the mRNA reads were associated with the functional category methane metabolism. The largest fraction of mRNA was from genes encoding components of the C1-carrier pathways [methyl-branch of the Wood-Ljungdahl pathway (Borrel et al., 2016; Adam et al., 2019)] and the methyltransferase system. Other abundant functional categories included important modules of methanogenesis, such as methyl-coenzyme M reductase, heterodisulfide reductase and formate dehydrogenase.

**Figure 4:**
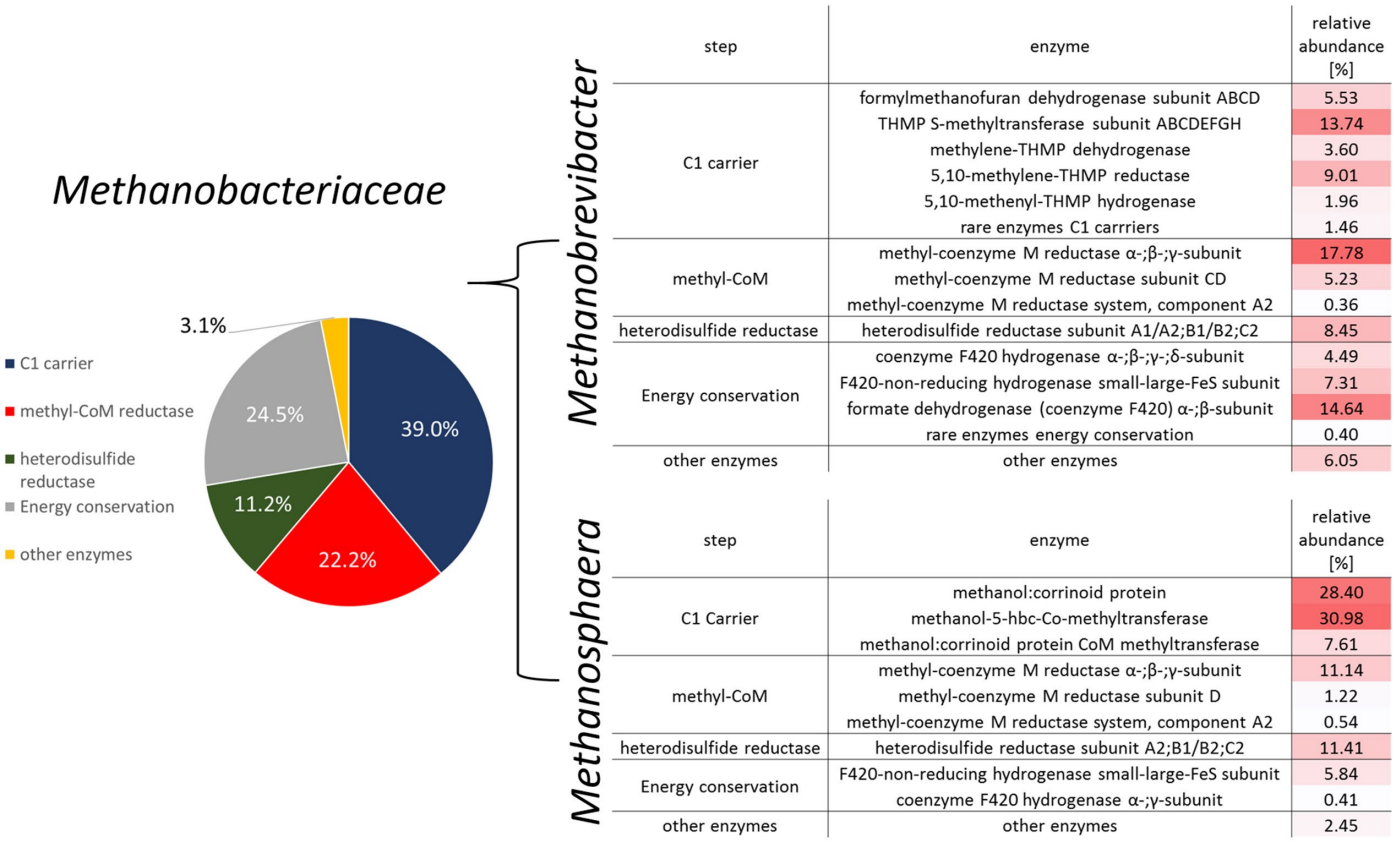
Overview of methane metabolism-related mRNA transcripts for the family *Methanobacteriaceae* (left) and a heatmap detailing the enzymatic distribution for the predominant archaeal genera *Methanobrevibacter* and *Methanosphaera* (right).

The analysis of the transcriptomes revealed that the type of methanogenesis was genus-specific. In accordance to literature (Liu and Whitman, 2008), the transcriptional pattern of the two dominant *Methanobrevibacter* species identified (*Mb. millerae* and *Mb. boviskoreanii*) indicated a hydrogenotrophic mode of methanogenesis (Figure 4). Transcripts of all main modules were rather abundant, such as those of proteins from electron donating reactions (i.e., formate dehydrogenase, coenzyme F420 dependent and coenzyme F420 independent hydrogenases). Transcripts of C1-carrier proteins and the core methanogenesis module consisting of *mcr* and heterodisulfide reductase comprised a substantial fraction as well. This is consistent with a hydrogenotrophic mode of methanogenesis and formate as an important electron donor for these dominant gut archaea (Rea et al., 2007).

Many fewer transcripts were obtained from the genus *Methanosphaera*. Here, 16S rRNA genes identified *Ms. cuniculi* as the dominant species, while *Ms. stadtmanae* was only detected at low abundances (Figure 2). Nevertheless, a methylotrophic mode of methanogenesis can be derived from the mRNA pattern (Figure 4). More than 60% of the transcripts originated from the methanol-specific methyl-group transfer complex consisting of methanol: corrinoid methyltransferase, the methanol-specific corrinoid protein and the methanol:corrinoid protein CoM methyltransferase. The *Mcr* as well as Hdr/Hyd complex were also well-represented among the transcripts (Figure 4). This is in accordance with a methylotrophic mode of methanogenesis, where methanol is being reduced to methane with electrons derived from hydrogen reduction (Biavati et al., 1988; Fricke et al., 2006; Söllinger et al., 2018). Similar to the observations in Figure 1, we were not able to identify genes of members of the order *Methanomassiliicoccales* in the metatranscriptomes of fecal samples.

#### 3.3.2. Signatures of IAV infection in archaeal proteome?

In the context of treatment-specific differences, Gierse and colleagues revealed that infection with the influenza A virus caused longitudinal alterations in the bacterial community composition, metaproteome and metabolome data within the samples of their animal trial. Predominant bacterial families were significantly reduced and specific enzymes involved in short-chain fatty acids (SCFA) synthesis were increased, a conclusion which was confirmed by the metabolome data (Gierse et al., 2021).

In supplemental findings, we have analyzed the specific archaeal proteome (2–3% of the total metaproteome) to illuminate previously unknown effects of an influenza A virus infection on structure and function of the archaeal community composition in swine gastrointestinal tracts. A list of the functional categories is included in Table 2. The output of the longitudinal scheme (from day 0 to day 31, see Table 1) was also included to provide a better overview of the study.

**Table 2:** Overview of the general functional categories of the identified archaeal proteome shown in relative abundance.

Category	Relative abundance [%]				Value of *p* (Kruskal-Wallis)	Significance code
	Naive	sd	H1N1	sd		
Energy production and conversion	24.85	4.49	19.51	3.50	0.05	*
Amino acid transport and metabolism	18.02	6.63	24.06	6.05	0.08	
Function unknown	12.79	4.53	15.49	2.68	0.18	
Coenzyme transport and metabolism	12.78	4.29	10.26	4.31	0.18	
Translation, ribosomal structure, and biogenesis	8.57	4.86	4.85	2.79	0.13	
Posttranslational modification, protein turnover, chaperones	7.00	3.23	4.93	1.70	0.08	
Carbohydrate transport and metabolism	3.48	0.82	4.27	1.37	0.21	
Unclassified	2.57	1.44	4.32	3.81	0.32	
Replication, recombination and repair	2.81	1.71	2.44	1.53	0.89	
Transcription	1.45	1.67	3.22	2.43	0.05	*
Lipid transport and metabolism	1.20	0.69	1.99	1.39	0.32	
Nucleotide transport and metabolism	1.75	1.47	0.93	0.42	0.21	
Cell wall/membrane/envelope biogenesis	1.05	0.58	1.29	0.84	0.32	
Inorganic ion transport and metabolism	0.90	0.44	1.07	0.62	0.32	
Defense mechanisms	0.46	0.43	0.34	0.29	0.60	
Signal transduction mechanisms	0.16	0.28	0.47	0.63	0.05	*
Secondary metabolites biosynthesis, transport, and catabolism	0.00	0.00	0.32	0.47	0.05	*
Cell cycle control, cell division, chromosome partitioning	0.15	0.34	0.12	0.38	0.61	
Intracellular trafficking, secretion, and vesicular transport	0.00	0.00	0.11	0.23	0.21	

(Mean values and standard deviation) and structured by the treatment [healthy (naïve, *n* = 3), infected (H1N1, *n* = 3)] and significance parameters (Kruskal-Wallis, *p* < 0.05).

Central functions of the archaeal metaproteome were significantly changed in the gastrointestinal tract of the H1N1 infected swine. In particular, proteins assigned to the functional categorie “energy production and conversion” was significantly reduced due to the infection. Conversely, levels of proteins involved in “transcription,” “signal transduction” and secondary metabolites biosynthesis increased significantly. Similar observations were described in the study of Gierse, in which clear alterations in the high abundant protein categories were evident in the early days following infection (Gierse et al., 2021).

A detailed analysis on predominant metabolic functions in the functional group “energy production and conversion” was the iron-containing metalloprotein Rubrerythrin (8.22% in healthy and 9.30% in infected). The second most abundant was a specific C1 carriage protein catalyzing the reduction of methylene H_4_MPT to methyl H_4_MPT, which had an average relative abundance of 8.67% in healthy and 2.42% in infected animals.

Despite these compelling signatures, the occurrence of archaeal protein groups in the metaproteome dataset was relatively minor [~2%, (Gierse et al., 2021)] compared to the presented 16S rRNA gene sequencing data, where archaea represent approximately 5–7% of the fecal community. However, their relative abundance might be overestimated with 16S rRNA gene sequencing due to potential amplification biases during PCR.

## 4. Conclusion and outlook

To our knowledge, this study is the first to describe a detailed pathway analysis of intestinal methanogenesis using comparative and integrative approaches of state of the art 16S rRNA gene sequencing, metatranscriptomics and metaproteomics. Ultimately, the study extended the knowledge of methanogens in the porcine intestinal tract by (1) providing insights into the dynamic composition of the archaeome along the intestinal tract, (2) verifying distinct methanogenesis pathways to the core archaea and (3) showing a minor impact of an IAV virus infection onto the swine archaeome. Focusing on temporal effects of the Influenza A virus infection, we observed that 5 of 12 phylotypes significantly changed between the naïve and H1N1 infected cohorts.

We identified different dominant phylotypes from both *Methanobrevibacter* and *Methanosphaera via* 16S rRNA gene sequencing. Then, we confirmed these observations with archaeal mRNA transcripts by assigning hydrogenotrophic and methylotrophic modes of methanogenesis to the predominant genera and phylotypes. Transcripts of all main methanogenesis modules were identified, and were found to be dominated by C1 carrier and methyl-coenzyme M reductase modules.

Furthermore, we described infection-induced signatures in highly abundant central metabolic functions within the archaeal proteome. Central metabolic functions, such as energy production, transcription, and signal transduction mechanisms, were significantly changed.

Despite the broad variety and volume of data generated by 16S rRNA gene sequencing, proteome and transcriptome techniques, the absolute quantity of methane formation in the intestines within the context of infections remains unknown and offers the opportunity for further investigations with focus on greenhouse gas emissions and livestock.

## Data availability statement

The datasets presented in this study can be found in online repositories. The names of the repository/repositories and accession number(s) can be found in the article/Supplementary material.

## Ethics statement

The animal study was approved by State Office for Agriculture, Food Safety and Fishery in Mecklenburg-Western Pomerania (LALFF M-V) with reference numbers 7221.3–1-035/17. The study was conducted in accordance with the local legislation and institutional requirements.

## Author contributions

AM and TU: conceptualization. AM, LG, CK, CW, PM, and BK: methodology. AM, LG, and CK: data curation. AM, LG, CK, VG, and HW, and TU: formal analysis. KR and TU: funding acquisition. AM and LG: investigation. TS, CS, BK, MB, DB, TM, KR, and TU: resources. AM: visualization. AM, LG, CK, and TU: writing–original draft. AM, LG, TS, CK, CS, DH, HW, VG, CW, PM, BK, MB, DB, TM, KR, and TU: writing–review and editing. All authors contributed to the article and approved the submitted version.

## Funding

This research was funded by Federal Excellence Initiative of Mecklenburg Western Pomerania and European Social Fund (ESF) Grant KoInfekt (ESF_14-BM-A55-0013_16, ESF_14-BM-A55-0006_16, ESF_14-BM-A55-0002_16, ESF_14-BM-A55-0008_16, and ESF_14-BM-A55-0010_16).

## Conflict of interest

The authors declare that the research was conducted in the absence of any commercial or financial relationships that could be construed as a potential conflict of interest.

## Publisher’s note

All claims expressed in this article are solely those of the authors and do not necessarily represent those of their affiliated organizations, or those of the publisher, the editors and the reviewers. Any product that may be evaluated in this article, or claim that may be made by its manufacturer, is not guaranteed or endorsed by the publisher.

## Footnotes

^1^ www.prophane.de
